# Solidified Milk

**Published:** 1855-05

**Authors:** 


### SOLIDIFIED MILK.

A committee of the New York Academy of Medicine recently visited the manufacturing of this important addition to culinary medicine. It must prove invaluable to travellers and especially to mothers living in cities where most of the milk is procured from diseased cows fed with distillery swill. We copy the following from the American Medical Monthly:

To 112 lbs. of milk, 28 lbs. Stuart's white sugar were added, and a trivial proportion of bicarbonate of soda, a tea-spoonful, merely enough to insure the neutralizing of any acid which in the summer season is exhibited even in a few minutes after milking, although inappreciable to the organs of taste. The sweet milk was poured into evaporating pans of enameled iron, embedded in warm water heated by steam. A thermometer was immersed in each of these water baths; that, by frequent inspection, the temperature may not rise above the point which years of experience have shown advisable.

To facilitate the evaporation—by means of blowers and other ingenious apparatus—a current of air is established between the covers of the pans and the solidified milk. Connected with the steam engine is an arrangement of stirrers, for agitating the milk slightly whilst evaporating, and so gently as not to *churn* it. In about three hours the milk and sugar assumed a paste consistency, and delighted the palate of all present; by constant manipulation and warming it was reduced to a rich, creamy looking powder, then exposed to the air to cool, weighed into parcels of a pound each, and by a press, with the force of a ton or two, made to assume the compact form of a tablet, (the size of a small brick,) in which shape, covered with tin foil, it is presented to the public.

Some of the solidified milk which had been grated and dissolved in water the evening previous, was found covered with a rich cream; this skimmed off, was soon converted into excellent butter. Another solution was speedily converted into wine whey, by a treatment precisely similar to that employed in using ordinary milk. It fully equalled the expectations of all; so that solidified milk will hereafter rank among the necessary appendages of the sick room. In fine, this article makes paps, custards, puddings, and cakes, equal to the best milk; and one may be sure it is an unadulterated article, obtained from well pastured cattle, and not the produce of distillery slops—neither can it be *watered.*

For our steamships, our packets, for those travelling by land or by sea, for hotel purposes or use in private families, for young or old, we recommend it cordially as a substitute for fresh milk.

